# Can the use of silver nanoparticles in dental implants increase its antimicrobial potency? - systematic review

**DOI:** 10.1186/s12903-025-06487-0

**Published:** 2025-07-19

**Authors:** Brice Joseph, Nessma Sultan, Soher Nagi Jayash

**Affiliations:** 1 Oral Biology department, Faculty of Dentistry, Mansoura University, Mansoura, 35516 Egypt; 2 Oral Biology and Dental Morphology, Faculty of Dentistry, Mansoura National University, Gamasa, 7731168 Egypt; 3 Roslin Institute, University of Edinburgh, Edinburgh, UK; 4 College of Medicine and Dentistry – Outreach Centre, Ulster University, Birmingham, UK

**Keywords:** Silver nanoparticles, Peri-implantitis, Dental implant biocompatibility

## Abstract

**Background:**

Silver nanoparticles (AgNPs) is a promising nanotechnological solution to prevent microbial colonization on dental implants, offering a promising avenue for combating implant-related infections and improving long-term treatment outcomes.

**Methods:**

Using manual search and electronic databases such as PubMed Advanced Search and Google Scholar, a methodical search was carried out from 2013 to 2023 utilizing keywords, and inclusion and exclusion criteria to find pertinent papers addressing the research question “Can the use of silver nanoparticles in dental implants increase its antimicrobial potency?”. Critical Appraisal analysis for this review was done using risk of bias tool for in vitro studies: QUIN tool.

**Results:**

Nine of the 122 articles that were retrieved underwent the three-step screening process before being chosen for the final analysis. QUIN Tool were used to evaluate the quality of the included in vitro studies. The summarized results from nine studies reveal the efficacy of AgNPs in inhibiting microorganisms and preventing biofilm formation on dental implants. In vitro tests demonstrate AgNPs’ ability to hinder bacterial growth associated with peri-implantitis. Various techniques, including surface modifications and nanostructure incorporations, exhibit promising antibacterial effects.

**Conclusions:**

AgNPs show promise in inhibiting multidrug-resistant bacteria, enhancing dental implant biocompatibility, and reducing peri-implant diseases, benefiting clinical outcomes and limiting bacterial colonization.

## Introduction

A breakthrough in implant research and technology over the past two decades has elevated implant-supported prostheses as the first line of therapy for long-lasting rehabilitation and the standard of care for replacing missing teeth [1] As a result, more edentulous patients, particularly those with severe bone insufficiency, are undergoing rehabilitation using current treatment protocols, which is related to an increase in peri-implant disorders. Silver (Ag) ions long been used in medical applications due to their potent antibacterial properties. Silver nanoparticles (AgNPs) possess Long-lasting bactericidal activity, low toxicity, excellent biocompatibility with cells from humans, and continual mobile ions to name a few [2]. Due to its potent antibacterial action against a variety of microbes, AgNPs can be used in dentistry to treat prophylactic infections in the mouth [3].

Nanotechnology has become a promising study field with multiple uses in science and technology. Structures within the nanoscale category have certain desired properties in biology and materials science [4]. AgNPs have been successfully used as antibacterial agents in a number of medicinal fields. AgNPs integration aims to eliminate microbial colonisation over dental biomaterials, which will also enhance overall oral health. Since ancient times, Ag ions have been used for their bactericidal properties. Even at low concentrations, they can demonstrate outstanding antibacterial characteristics because of their nanoscale size and significant surface-to-volume ratio [5].

AgNPs’ ability to combat clinical dental biofilms linked to disorders affecting dental implants has not yet been determined. However, due to a lack of sufficient data, it is vital to find a new therapy to combat the overgrowth of harmful microorganisms in implants. Therefore, the purpose of this study is to assess how well AgNPs can stop bacteria from growing around dental implants.

Researchers are attempting to use the antibacterial properties of AgNPs in dentistry by incorporating them into a variety of dental materials, including resin composites for direct restorations, adhesives, acrylic resins that are utilised for denture fabrication, guided tissue regeneration membranes, irrigation materials, and obturation materials in endodontics. According to the study by Bapat, Chaubal [6], the application of AgNPs to titanium dental implants can lessen the incidence of peri-implant disorders. The study reported that titanium implants coated with AgNPs have better increased osteogenic potential and better cytocompatibility. According to evidence presented by García-Contreras, Argueta-Figueroa [7], the quantity of free Ag ions released into the medium was directly correlated with the concentration of AgNPs.

There is fear that AgNPs could enter cells like a Trojan horse, release Ag ions, and disrupt normal cell activity. According to certain laboratory investigations, AgNPs may cause oxidative stress and compromise mitochondrial function in human cells [8]. Additionally, a number of in vitro studies indicate that AgNPs are hazardous to mammalian cells from the vascular, reproductive, skin, brain, liver, and lung systems [9]. Numerous studies have shown that various metallic nanoparticles can result in decreased cell survival rates, reactive oxygen species formation, mitochondrial damage, strands of DNA breaks, autophagy, or pyroptosis, apoptosis, and various other types of cell death [10]. Since the evidence is still preliminary, additional investigations examining in vitro toxicity as well as lengthy in vivo studies concentrating on mutagenicity and cytotoxicity should be conducted to guarantee the clinical safety of these particles. Hence, this review aims at having an indebt literature about the antimicrobial effects of AgNPs and its success when used in dental implants.

The rationale for conducting this systematic review arose from the antimicrobial resistance (AMR) which is a public health concern [11]. Implant failure can be a result of the degeneration of supporting bone caused by inflammation which can influence the tissues encircling an osseo-integrated implant due to the host tissue being unable to start or keep up osseointegration [12]. Bacterial plaque or bacterial biofilms can be attributed as being the primary risk factors for gingivitis and periodontitis, which can sequentially cause implant failure [13].

## Materials and methods

### Research question

Can the use of silver nanoparticles in dental implants increase its antimicrobial potency?. This research has the intention of advising professionals whether the use of AgNPs in dental implant has better antimicrobial effectiveness. To answer the above research question, the author used the Population, Exposure, Outcome (PEO) framework formula; population: dental Implants, exposure: use of AgNPs and outcomes: increased antimicrobial potency.

### Study design

This study will be carried out as a systematic review of studies to evaluate the efficiency of AgNPs in reducing the colonization of microbes when in contact with dental implants.

### Literature search strategy

Published material, including original articles and research papers, on the use of AgNPs in dental implants can be found in the electronic databases PubMed, Google Scholar and Science Direct will be used in the search protocol with a time limit from 2013 to 2023. The search approach will integrate medical search terms (MeSH) and keywords with Boolean operators: “silver nanoparticles OR silver nanostructure OR AgNP OR AgNPs AND dental implant OR dental implants OR implants AND antibacterial OR antimicrobial OR antibiofilm”.

#### Selection criteria

The inclusion criteria included were as the follow; (1) Original publications that have been published during the past 10 years (2013 to present), (2) Studies published in English, (3) Clinical and preclinical studies, (4) Full-text articles, (5) Relation to implant dentistry, (6) Focus on silver nanoparticles on dental implants. The exclusion criteria were; (1) Case report or conference paper, (2) Only abstracts are available, (3) Unrelated articles.

### Literature screening stages

**Fig. 1:**
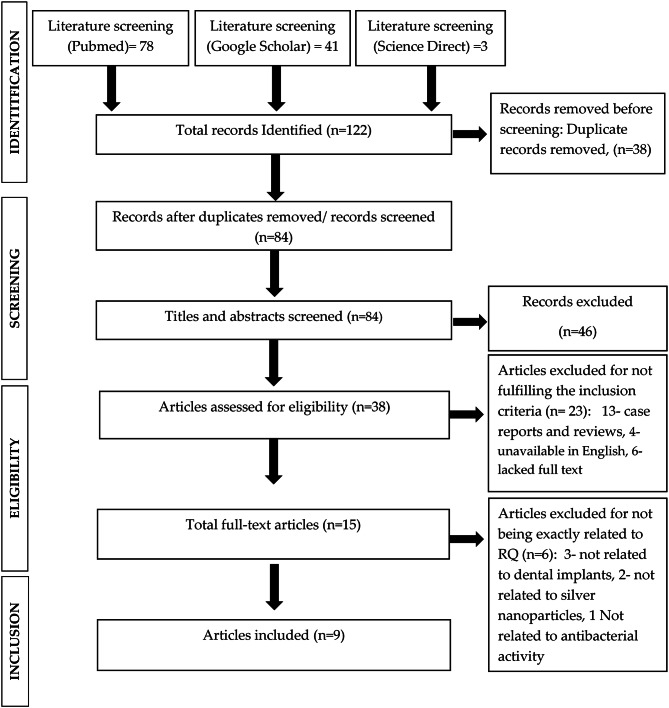
PRISMA Flowchart to the screening process

The Preferred Reporting Items for Systematic Reviews and Meta-analyses (PRISMA) checklist was used to identify the articles that will be included in this review (Fig. 1). Upon doing an initial search and implementing filters via three screening phases, the studies which were finally chosen for assessment were located. The articles were chosen by using the PRISMA flow chart as a guide. The first step was addressing the study issue by reading the article titles and reviewing their applicability. Reading the article abstracts in order to determine which ones were appropriate to read the full text of was the second step. The third stage was finished by thoroughly reading each article and using the inclusion and exclusion criteria.

### Quality assessment

Depending on the type of study, various tools was used for the critical evaluation of research, such as the QUIN tool for in vitro experiments. The QUIN Tool (quality assessment tool for in vitro research) was used to obtain and critically review the full texts of the studies that meet the inclusion criteria (Sheth et al., 2022).

### Synthesis of findings

Finding out how efficient AgNPs are as antimicrobials when added to dental implants was the main outcome measure. In the discussion part, the results of the various investigations were presented. Regarding the function of AgNPs in dental implants, the key conclusions, advantages, and disadvantages of the chosen studies were emphasised and thoroughly examined.

## Results

### Study selection

In the initial search using key search terms identified 122 articles, with 78 studies in PubMed search engines, 41 studies in Google Scholar search engines and 3 studies from Science Direct, indicating that principal search strategies was effective. After filtering, 9 articles that deemed to satisfy the acceptable requirements of inclusion criteria were included after the third level of screening (Fig. 1).

### Study characteristics

The results of the 9 studies included are summarized in the Table 1 given below. These selected studies demonstrate how well AgNPs inhibit microorganisms and stop biofilm formation on dental implants. A study by El-Telbany and El-Sharaki [14] demonstrated that AgNPs successfully countered multidrug resistance by inhibiting Aeruginosa growth highlighted how AgNPs may be used to destroy these biofilms. Ag-chitosan nanoparticles antibacterial activities against dental implant microorganisms were revealed by Divakar, Jastaniyah [15]. emphasising on how incorporating AgNPs might improve implant corrosion resistance, lessen cytotoxicity, and increase biocompatibility. Besinis, Hadi [16]. investigated the important antibacterial and antibiofilm properties of silver-titanium dioxide-hydroxyapatite nanocoatings. highlighted the capacity of a two-layered silver-HA nanocoating to stop the growth of germs without sacrificing HA’s biocompatibility, which is essential for bone repair. Choi, Jang [17]. Demonstrated the effectiveness of polydopamine and silver-coated titanium in delaying microbial growth and averting the formation of biofilms, which are crucial in impeding the pathogenesis of gum disease. The capacity of AgNPs to lessen Candida albicans colonisation into external hexagon implants has been shown by Matsubara, Igai [18]. Guo, Cui [19]. investigated the induction of mineralization and antibacterial activity of a composite coating, highlighting the coating’s modest cytotoxicity and capacity to prevent bacterial colonisation while encouraging mineralization. A hydrogen titanate nanotube layer containing AgNPs was presented by Wang, Sun [20]. as a bacteriostatic and biocompatible implant material that exhibited strong antibacterial capability without causing appreciable harm. The antibacterial characteristics of titanium implants doped with nano silver were studied by Pokrowiecki, Zaręba [21]. in relation to biofilm bacteria that grow around implants noticing its strong antibacterial properties. Venugopal, Muthuchamy [22]. investigated titanium micro implants treated with AgNPs, which demonstrated remarkable antibacterial qualities. These findings illuminate AgNPs’ potential in enhancing implant biocompatibility, reducing microbial contamination, and mitigating peri-implant diseases, showcasing their role in advancing implant materials for improved clinical outcomes and reduced bacterial colonization.

### Critical appraisal and risk of bias in individual studies

Table 2 shows the overall results of the risk of bias assessment of the 9 studies. Since all the studies included are in vitro, QUIN tool was used to assess the risk of bias. All the studies give a distinct direction by clearly defining their goals and objectives. Methodology explanations of the included studies are thorough, ensuring that their processes and strategies are transparent. All studies give adequate explanations for sample size figures, but the characterizations of sampling methods vary. Most studies included provide adequate information on comparison groups, which raises the standard of their comparative analysis. The fact that blinding, outcome assessor specifications, operator details, and randomization are all identified as not relevant across all research suggests that the approaches used were not given enough thought or significance. Every study uses a well-defined approach for measuring outcomes to guarantee the validity of the data they have analysed. However, there are mistakes in the thorough explanation of statistical analysis. A few studies lack specificity, which could jeopardise the validity of their statistical findings, even though many studies sufficiently describe their statistical methodology. Overall, the way the results are presented is sufficient to ensure that their conclusions and observations are understood. In conclusion, there are discrepancies in the descriptions of sampling techniques and in-depth statistical analyses, even if the majority of studies perform well in defining goals and objectives, outlining methodology, and presenting results. These differences point out areas that should be improved upon to increase the scientific rigour and validity of the studies’ conclusions.

**Table 1:** Main characteristics of included in vitro studies

Sr. No.	Author/Year	Technique	Microbial Specimen	Result	Significance
1	El-Telbany and El-Sharaki [14].	Specimens from patients with peri-implantitis were taken for isolation of *p. aeruginosa*. DNA was extracted from the isolates and 16 S rDNA-based PCR assay was used to confirm the identification. Susceptibility of isolated *p. aeruginosa* to 16 antibiotics was evaluated using the VITEK 2 system. The growth inhibition of isolated bacteria by AgNPs was tested by disk-diffusion method. The microtiter plate assay was used to estimate the capacity of *P.* aeruginosa to from biofilms. Antibiofilm activity of AgNPs was determined by microtiter plate assay	*P. aeruginosa*	*P. aeruginosa* isolates were resistance to most of used antibiotics. All tested concentration of AgNP_S_ exhibited a greatest anti-biofilm activity against multi-drug resistance p. aeruginosa	Current findings highlight the role of AgNP_S_ in growth inhibition of P. aeruginosa and reveal a potential application of AgNPS in eradication of *p. aeruginosa* biofilms.
2	Divakar, Jastaniyah [15].	Bacterial species inoculated in 24-well plates at continuous agitation for up to 12 h at 37 °C in a CO2 incubator for *S. mutans* and anaerobically (*P. gingivalis*), Ag nanoparticles conjugated with chitosan, purified chitosan, and gentamicin as standard were added. Control without inoculum, inoculum without any treatment, Ag conjugated with chitosan, purified chitosan, Ag-chitosan and antibiotics in combination with gentamicin were used to compare the results and maintained simultaneously. After the culture was completed in a spread plate both before and after sonication, the average colony forming units were determined.	*P. gingivalis* & *S. mutans*	The antibacterial activity of the Ag-chitosan combination is greater against the test organism and had a sound inhibitory effect on the growth of bacteria.	Coating of titanium dental implants with Ag-chitosan may have an added advantage on the corrosion resistance of dental implants and also augments the passivating effect of these implants.
3	Besinis, Hadi [16]	Titanium alloy implants were surface modified to produce a mixture of silver, titanium dioxide, and hydroxyapatite (HA)nanocoating. The quantitative evaluation of their antibacterial efficacy involved monitoring the growth of *Streptococcus sanguinis*, the percentage of living and dead cells, and the lactate production of the microorganisms over a 24-hour period.	*Streptococcus sanguinis*	A significant antibacterial and antibiofilm activity for each of the three silver plated groups (Ag, Ag + nano-hydroxyapatite, and Ag + micro-hydroxyapatite), with the silver plated discs coated with nano-hydroxyapatite. A dual-layered silver-HA nanocoating was applied to the surface of implants, bacterial growth in the surrounding media was effectively prevented (100% mortality), and bacterial biofilm development on the implant surfaces was decreased by 97.5%. Titanium dioxide nanocoating and uncoated controls did not exhibit any antibacterial activity.	By applying a two-layered silver- hydroxyapatite nanocoating on titanium alloy implants, antibiofilm qualities are created on the surface without sacrificing the biocompatibility of hydroxyapatite, which is necessary for osseointegration and faster bone repair.
4	Choi, Jang [17].	Silver-loaded poly dopamine coating was formed by immersing pure titanium in dopamine hydrochloride/ HCl buffer solution for 24 h in 50mL silver nitrate solutions with different concentrations for 30 min. Microbial growth inhibition and microbial growth curve analyses for bacterial solutions of *S. mutans* and *P. gingivalis* incubated with the specimens were respectively conducted by counting the numbers of colonies on agar solid medium and by measuring absorbance using enzyme-linked immunosorbent assay reader.	*Streptococcus mutans* and *Porphyromonas gingivalis*	AgNPs were uniformly distributed over the whole surface of the polydopamine and silver coated titanium specimens. The numbers of microbial colonies for both bacteria cultured with surface-modified titanium were significantly lower than those cultured with uncoated titanium. When *Streptococcus mutans* and *Porphyromonas gingivalis* were cultured with surface-modified titanium, the lag phase of the growth curves for both bacteria were continually maintained, whereas the lag phase for *streptococcus mutans* and *porphyromonas gingivalis* changed to exponential phase after 9 and 15 h, respectively, when both bacteria were cultured with uncoated titanium.	It was confirmed that the coating of polydopamine and silver on the surface of titanium effectively retards the microbial growth, which can cause the formation of biofilm and pathogenesis of gum disease in the mouth.
5	Matsubara, Igai [18].	*Candida albicans* grown in Sabouraud dextrose broth was used. Three groups made up the in vitro model: a negative control group that received sterile SDB, a positive control group that received sterile phosphate-buffered saline, and an experimental group that applied nano-Ag suspension. Abutments for temporary crowns and external hexagon implants were used. Implant/abutment units were submerged in C. albicans suspension, allowed to incubate, and then examined for fungal contamination as part of the microbiological investigation process.	*Candida albicans*	The experimental group had substantially less *C. albicans* colonisation than the positive control group. This decrease showed that, during a 72-hour exposure to a *C. albicans* suspension, nano-Ag was effective in preventing the growth of C. albicans within implants.	The use of AgNPs within external hexagonal implants successfully reduces the colonisation of Candida albicans on their internal surfaces.
6.	Guo, Cui [19].	The purpose of this study was to prepare a composite coating on a porous titanium surface for infection prevention and inducing mineralization, which was initialized by deposition of a poly-L-lysine /sodium alginate/ PLL self-assembled coating, followed by dopamine deposition, and finally in situ reduction of silver nanoparticles (AgNPs) by dopamine. *S. mutans* and *S. aureus*, were used to assess the antibacterial qualities of the samples.	*Streptococcus mutans* and *Streptococcus aureus*	Ag was released over a minimum of 27 days. Following *S. aureus* and *S. mutans* incubation, pTi/PSP/DA/Ag may inhibit bacterial colonisation and adherence. The composite coating shown acceptable cytocompatibility after being incubated in SBF, but it also displayed mild cytotoxicity that prevented cell attachment. This coating could cause mineralization on the surface.	The composite coating could prevent bacterial infections and facilitate mineralization in vivo in the early postoperative period, and then, the mineralized surface could enhance the cytocompatibility.
7.	Wang, Sun [20].	Using an alkali-hydrothermal process and HCl treatment which can facilitate the integration of the implant directly with the surrounding bone structure. By absorbing silver nitrate solution, Ag ions are introduced into the nanotubes. Gram-negative *Escherichia coli* DH5α (*E. coli*) was cultivated in Luria-Bertani (LB) broth under 200 rpm shaking at 37 °C for 3 h to assess the antimicrobial properties of the three samples. After coincubation in a rotary shaker at 37 °C for 24 h, 100 µL of culture suspension from each tube was uniformly spread on the LB agar plates and the number of viable bacterial colonies was counted after incubation at 37 °C for 24 h	*Escherichia coli*	The tube test directly demonstrated that both sandwich nanostructures effectively inhibit bacteria growth. To quantify the tube test results, the bactericidal rates of AgNO3-NT-Ti and AgNP-NT-Ti samples were 99.994% and 99.987%, respectively	Antimicrobial and biocompatible tests have shown that the sandwich nanostructure with a low level of silver loading exhibits a bacteriostatic rate as high as 99.99%, while retaining low toxicity for cells and possessing high osteogenic potential. Titanium foil with a AgNPs filled hydrogen titanate nanotube layer on the surface that is fabricated with low-cost surface modification
8.	Pokrowiecki, Zaręba [21].	Titanium disks were incorporated with AgNPs over different time periods by Tollens reaction. The surface roughness, wettability, and silver release profile of each disk were measured. In addition, the antibacterial activity was also evaluated by using disk diffusion tests for bacteria frequently isolated from the peri-implant biofilm: *Streptococcus mutans*, *Streptococcus mitis*, *Streptococcus oralis*, *Streptococcus sanguis*, *Porphyromonas gingivalis*, *Staphylococcus aureus*, and *Escherichia coli*. Cytotoxicity was evaluated in vitro in a natural human osteoblasts cell culture.	*Streptococcus mutans*, *Streptococcus mitis*, *Streptococcus oralis*, *Streptococcus sanguis*, *Porphyromonas gingivalis*, *Staphylococcus aureus*, and *Escherichia coli*	Ag containing disks provide sufficient antibacterial properties compared to K1 and K2 disks. Gram-negative bacteria were more susceptible to nanosilver than Gram-positive bacteria. The inhibition of *P. gingivalis* was most pronounced, in comparison with other species except *E. coli*. In the group of Gram-positive bacteria, *S. aureus* was the most susceptible to AgNPs. The Zone of Inhibition of these bacteria was significantly wider than those of *S. mutans* and *Streptococcus oralis*. In the Streptococcal subgroup, *S. sanguis* exhibited the largest inhibition zones, whereas *S. mutans* was the most resistant to AgNPs	nanosilver on the titanium provides an antibacterial activity involved in peri-implantitis.
9.	Venugopal, Muthuchamy [22].	AgNP-modified titanium microimplants (Ti-nAg) were prepared using two methods. The first method involved coating the microimplants with regular AgNPs (Ti-AgNP) and the second involved coating them with a AgNP-coated biopolymer (Ti-BP-AgNP). The topologies, microstructures, and chemical compositions of the surfaces of the Ti-nAg were characterized by scanning electron microscopy (SEM) equipped with energy dispersive spectrometer and X-ray photoelectron spectroscopy.	*Streptococcus mutans*, *Streptococcus sanguinis*, and *Aggregatibacter actinomycetemcomitans*	Antibacterial activity of the micro implants was confirmed by the presence of an inhibition zone of the growth of *S. mutans*, *S. sanguinis*, and *A. actinomycetemcometans* around the substrates. Bacterial growth was detected around both the control as well as the Ti-AgNP microimplants. No bacterial growth was observed around. the Ti-BP-AgNP microimplants.	Titanium micro implants modified with Ti-BP-AgNP exhibit excellent antibacterial properties, making them a promising implantable biomaterial.

**Table 2:** Summary of risk of bias for in vitro studies using QUIN TOOL

Criteria No	Criteria	[20].	[22].	[21].	[17].	[14].	[19].	[15].	[16].	[18]
1	**Clearly stated aims/objectives**	Adequately specified	Adequately specified	Adequately specified	Adequately specified	Adequately specified	Adequately specified	Adequately specified	Adequately specified	Adequately specified
2	**Detailed explanation of sample size calculation**	Adequately specified	Adequately specified	Adequately specified	Adequately specified	Adequately specified	Adequately specified	Adequately specified	Adequately specified	Adequately specified
3	**Details of comparison group**	Adequately specified	Adequately specified	Adequately specified	Adequately specified	Adequately specified	Adequately specified	Adequately specified	Adequately specified	Adequately specified
4	**Detailed explanation of methodology**	Adequately specified	Adequately specified	Adequately specified	Adequately specified	Adequately specified	Adequately specified	Adequately specified	Adequately specified	Adequately specified
5	**Detailed explanation of sampling technique**	Adequately specified	Adequately specified	Adequately specified	Adequately specified	Adequately specified	Adequately specified	Adequately specified	Adequately specified	Adequately specified
6	**Operator details**	Not applicable	Not applicable	Not applicable	Not applicable	Not applicable	Not applicable	Not applicable	Not applicable	Not applicable
7	**Randomization**	Not applicable	Not applicable	Not applicable	Not applicable	Not applicable	Not applicable	Not applicable	Not applicable	Not applicable
8	**Method of measurement of outcome**	Adequately specified	Adequately specified	Adequately specified	Adequately specified	Adequately specified	Adequately specified	Adequately specified	Adequately specified	Adequately specified
9	**Outcome assessor details**	Not applicable	Not applicable	Not applicable	Not applicable	Not applicable	Not applicable	Not applicable	Not applicable	Not applicable
10	**Blinding**	Not applicable	Not applicable	Not applicable	Not applicable	Not applicable	Not applicable	Not applicable	Not applicable	Not applicable
11	**Statistical analysis**	Adequately specified	Adequately specified	Adequately specified	Adequately specified	Adequately specified	Adequately specified	Not specified	Adequately specified	Adequately specified
12	**Presentation of results**	Adequately specified	Adequately specified	Adequately specified	Adequately specified	Adequately specified	Adequately specified	Adequately specified	Adequately specified	Adequately specified

## Discussion

Dental implants have been utilised for a number of purposes, including aesthetic therapy and the recovery of masticatory capabilities. Infections associated to implants, like peri-implant mucositis and peri-implantitis, are frequent side effects in the dental field [23]. Surface characteristics as well as implant-related factors can increase the likelihood of peri-implant disease [24]. Therefore, the goal of numerous investigations has been to create implant surfaces that are antibacterial. Among these techniques is the application of AgNPs. A study by Yin, Zhang [25]. demonstrated that Ag possesses a wide range of antibacterial properties, which are achieved by interfering with the permeability of bacterial cell walls, causing damage to DNA, and rendering vital proteins inactive. The research that are included have looked at the antibacterial activity against various types of bacteria. Regarding the antimicrobial effect, they often get satisfactory results. Zhai, Tian [26]. emphasised that a wide range of techniques could be used to alter implant surfaces in an effort to increase their antibacterial power while maintaining enhanced biocompatibility. The adaptability of AgNPs in fighting bacterial infections was demonstrated by researchers who experimented with a variety of tactics, such as conjugating AgNPs with chitosan or inserting them into different nanocoating’s [15]. AgNPs have demonstrated consistent antibacterial efficacy against a variety of microorganisms that are commonly linked to peri-implant disorders, such as *Candida albicans*, *Pseudomonas aeruginosa*, *Streptococcus mutans*, and *Porphyromonas gingivalis* [14, 15, 18]. which in turn can support AgNPs’ potential as a potential antibacterial agent for dental implants. Besinis, Hadi [16] and Guo, Cui [19] demonstrated the effectiveness of additional surface changes in addition to AgNPs. The outcomes showed that these modified surfaces had strong antibacterial activity and inhibited the production of biofilms.

Significantly, these researches included the biocompatibility component, highlighting the necessity of ensuring compatibility with the host tissue in addition to fighting bacterial colonisation. A study conducted by Wang, Sun [20] showed minimal cytotoxicity and maintained or even enhanced biocompatibility with of these modified surfaces. This is an essential aspect in ensuring that the adapted implants not only guard against infections but also encourage positive tissue reactions, which support effective long-term implant integration.

Despite the promising outcomes, Venugopal, Muthuchamy [22] and Matsubara, Igai [18]. emphasised about potential cytotoxicity risks related with specific surface changes. Throughout these studies, there was a constant emphasis on the need for more research into the long-term impacts, possible side effects, and ideal doses of antibacterial agents like AgNPs. This emphasises how important it is to have a balanced approach in clinical applications to ensure safety and long-term biocompatibility without compromising antibacterial activity.

To sum up, the variety of techniques used to alter implant surfaces highlights the ever-changing field of study focused on boosting antibacterial qualities while preserving or enhancing biocompatibility. Consistent attention to AgNPs combined with a variety of coating materials and techniques shows encouraging progress in creating surfaces for implants that not only successfully prevent bacterial colonisation but also create an environment that is favourable for osseointegration. However, further investigations into the long-term effects, optimal concentrations, and potential toxicity of these modifications are crucial to translate these findings into safe and efficacious clinical applications.

### Limitation and future research suggestion

The limitations observed across current studies on dental implants incorporating silver nanoparticles highlight key gaps in our understanding of their clinical relevance. Most existing investigations are conducted in vitro, which poses challenges in accurately replicating the complex conditions of the oral environment. Therefore, in vitro findings must be validated through in vivo studies and clinical trials to better assess the real-world efficacy and long-term antimicrobial performance of AgNP-coated implants.

Future research should focus on:

Evaluating a broader spectrum of relevant oral pathogens;

Conducting long-term studies on biocompatibility, cytotoxicity, and systemic effects;

Performing detailed surface property characterization of AgNP-functionalized implants.

These efforts will be essential to bridge the gap between laboratory results and clinical applications, ultimately enhancing the reliability, safety, and practical value of silver-based dental implant technologies.

## Conclusions

Current research suggests that incorporating AgNPs into dental implant materials may offer potential benefits in reducing bacterial infections, inhibiting biofilm formation, and supporting conditions favourable for implantation. However, further experimental and clinical studies are essential to confirm these findings and to establish safe and effective strategies for integrating AgNPs into dental materials. Given the complexity of their antimicrobial activity and variability in performance, continued investigation is needed to better understand their role and optimize their use in preventing peri-implantitis and other implant-related infections.

## Data Availability

Data are available within the manuscript.

## Abbreviations

AgNPs: Silver nanoparticles
Ag: Silver
AMR: Antimicrobial resistance
PEO: Population, exposure, outcome
PRISMA: Preferred reporting items for systematic reviews and meta-analyses
pTi/PSP/DA/Ag: Polished titanium/ polysaccharide-based coating/ dopamine/ sliver
Streptococcus mutans: S. mutans
Porphyromonas gingivalis: P. gingivalis
Candida albicans: C. albicans
Ti-BP-AgNP: Titanium- bisphosphonate- silver nanoparticles
BP: Bisphosphonate
